# A Multi-Sectoral Approach Improves Early Child Development in a Disadvantaged Community in Peru: Role of Community Gardens, Nutrition Workshops and Enhanced Caregiver-Child Interaction: Project “Wawa Illari”

**DOI:** 10.3389/fpubh.2020.567900

**Published:** 2020-11-06

**Authors:** Doris González-Fernández, Ana Sofía Mazzini Salom, Fermina Herrera Bendezu, Sonia Huamán, Bertha Rojas Hernández, Illène Pevec, Eliana Mariana Galarza Izquierdo, Nicoletta Armstrong, Virginia Thomas, Sonia Vela Gonzáles, Carlos Gonzáles Saravia, Marilyn E. Scott, Kristine G. Koski

**Affiliations:** ^1^School of Human Nutrition, McGill University, Sainte Anne de Bellevue, QC, Canada; ^2^International Child Development Program - ICDP Peru, Lima, Peru; ^3^Pachacámac Health Center, Ministry of Health, Lima, Peru; ^4^Laboratory School, Faculty of Medical Technology, Federico Villarreal National University, Lima, Peru; ^5^Community Engagement, Design and Research Center (CEDaR), University of Colorado, Boulder, CO, United States; ^6^“Vivir” Association, Quito, Ecuador; ^7^International Child Development Program (ICDP), Oslo, Norway; ^8^Susila Dharma International Association, Greenfield Park, QC, Canada; ^9^Nursing School, Inca Garcilaso de la Vega University, Lima, Peru; ^10^Dermatology Service, Department of Medicine, National Institute for Children's Health (INSN), Lima, Peru; ^11^Institute of Parasitology, McGill University, Sainte Anne de Bellevue, QC, Canada

**Keywords:** monitoring, learning system, infant development, diet diversity, food secuity, language development, caregiver-child interaction, home gardens

## Abstract

**Background:** Multi-dimensional monitoring evaluation and learning strategies are needed to address the complex set of factors that affect early child development in marginalized populations, but few studies have explored their effectiveness.

**Objective:** To compare improvement of health and development of children 0–3 years between intervention communities (IC) and control communities (CC) from peripheral settlements of Lima. Sequential interventions included: (1) home and community gardens, (2) conscious nutrition, and (3) parenting workshops following the International Child Development Program (ICDP).

**Methods:** Interventions were delivered by community health promoters (CHPs) using a “step-by-step” learning system. Both IC and CC were monitored before the interventions began, at 8 and 12 months (*n* = 113 IC and 127 CC children). Data were collected on household characteristics, diet, food security, health indicators (history of diarrhea and respiratory infections, hemoglobin, intestinal parasites, anthropometry), caregiver-child interactions and stress, and achievement of Pan-American Health Organization age-specific developmental milestones. Stepwise multiple logistic regressions were used to determine if the interventions affected food insecurity, as well as motor, social/cognitive and language delays.

**Results:** At baseline, 2.6% were categorized as “suspected developmental delay” and 14.2% were on “alert for development delay.” Food insecurity, diarrhea and respiratory infections were lowered following the interventions. Through the “step-by-step” approach, caregivers in IC gained skills in gardening, conscious nutrition and parenting that reduced the risk of food insecurity [Adjusted Risk Ratio = 0.20 (95% CI: 0.08–0.51)] and language delay [0.39 (0.19–0.82)] but not motor or social/cognitive delay. Use of a multiple micronutrient supplement decreased the risk of motor delay [0.12 (0.03–0.56)], but more pets were associated with higher risk of motor [3.24 (1.47–7.14)] and social/cognitive delay [2.72 (1.33–5.55)], and of food insecurity [1.73 (1.13–2.66)].

**Conclusion:** The combined interventions delivered by CHPs helped to mitigate the impact of adversity on food insecurity and language delay. Additional improvements may have been detected if the interventions had continued for a longer time. Our results indicate that control of infections and pets may be needed to achieve measurable results for motor and social/cognitive development. Continuous monitoring facilitated adjusting implementation strategies and achieving positive developmental outcomes.

## Introduction

The World Health Organization estimates that in any country about 10% of the population has some type of disability or developmental delay (1), and based on growth retardation and poverty measures, an estimated 43% of children under 5 are at risk of not reaching their development potential (2). Surveys of 35 low and middle-income countries have reported that 14.6% of children have low scores in the early development index associated with cognition, 26.2% have low socio-emotional scores, and 36.8% have both conditions (3). It is projected that children at risk of developmental delay due to growth problems and poverty will have a quarter of the average income of one adult per year, and that the cost of lack of action to the net domestic product can become twice as much as those countries that invest in health (4). Thus, the decline in growth retardation, the primary measure of chronic malnutrition (5), is considered a global priority, and a call has been made to investigate possible cost-effective and scalable interventions for the transition to the Sustainable Development Goals post-2015 (6). Current evidence on risk factors for developmental delay in early childhood in low-income areas includes a number of preventable factors that can alter the course of neurodevelopment including nutrition, infectious diseases and inflammation, caregiver insensitivity and chronic stress (7).

Food insecurity influences child development through its effects on nutrition and by producing stress in the family (8). Small children achieve satiety with cheap foods like sweetened liquids and junk food, but the total intake of macro and micronutrients may be insufficient for normal growth (8). Interventions with home and community gardens constitute an important strategy to improve food security, and particularly in the Andean region, provide the opportunity to grow traditional products with high nutritional value (9). Interventions that include creating home gardens and the consumption of traditional regional foods have shown improved food diversity in infants undergoing complementary feeding (10) for growth and achieving adequate weight and height (11).

Both protein energy malnutrition and/or infections produce not only growth retardation, but also structural and functional brain damage that presents as a delay in the development of cognitive functions and permanent cognitive damage (12). Among specific nutritional deficiencies, anemia as a manifestation of iron deficiency is one of the main causes of brain damage and delay in cognitive, behavioral and psychomotor development (13). However, nutrition-focused interventions have not always been successful. This is believed to be due to the inflammation caused by infections or environmental enteropathy, largely associated with the absence of clean drinking water or adequate sanitation and hygiene (14, 15). In fact, interventions that include water improvement, sanitation and hygiene have been shown to have a positive effect not only in the reduction of acute diarrheal disease, but also in respiratory infection, intestinal parasitism and other infectious diseases, with a final result of improvement in the anthropometry of children (16).

Recent research has demonstrated the importance of the caregiver-child relationship in child development, finding, for example, that harmonious, reciprocal interactions that reflect an emotional relationship evidenced by emotional and/or verbal sharing, improve cognitive and mental development and children's language (17). It is known that the practice of verbal and non-verbal attitudes by the caregiver to the child during early stages of child's development is able to modulate the expression of language systems (18). On the other hand, an inadequate caregiver-child interaction may result in eating disorders, physical under-development, decreased attention and cooperation, and an inability to learn and to develop adequate interpersonal relationships (19). Moreover, it has been documented that infants of caregivers who used positive parenting skills had greater increases in language production (20), and that characteristics of caregivers can influence the nutritional status of the child, even when controlling for the socio-economic status (21).

The first 1,000 days of life constitute an important window to improve health conditions in early childhood and in mothers to prevent the cycle of inter-generational delay (22). A recent analysis of strategies aimed at improving the development of children, shows that in order to achieve adequate development of children, multi-sectoral interventions are required in health, nutrition, education and child protection (23).

The goal of this project was to explore whether a multi-dimensional approach to early child development that emphasized home gardens, nutrition and improved caregiver-child interaction in a context of ongoing adaptation to local conditions would demonstrate results in a marginalized community in a relatively short 18 month time frame. The project was structured as both a field intervention for early child development and a longitudinal study to shape and describe the impact on health and development of children 0–3 years from “Centros Poblados Rurales” (CPRs) of Pachacámac (Peru), through participation of community health promoters (CHPs) and the local Ministry of Health. The Wawa Illari (“Wawa = child, Illari = resplendent” in Quechua) project consisted in the combination and ongoing adaptation of three interventions: (1) creation of community and home gardens, (2) workshops in conscious nutrition and meal preparation, and (3) improvement of the caregiver-child interaction, compared to the standard intervention.

## Methods

### Study Design

**Figure 1:**
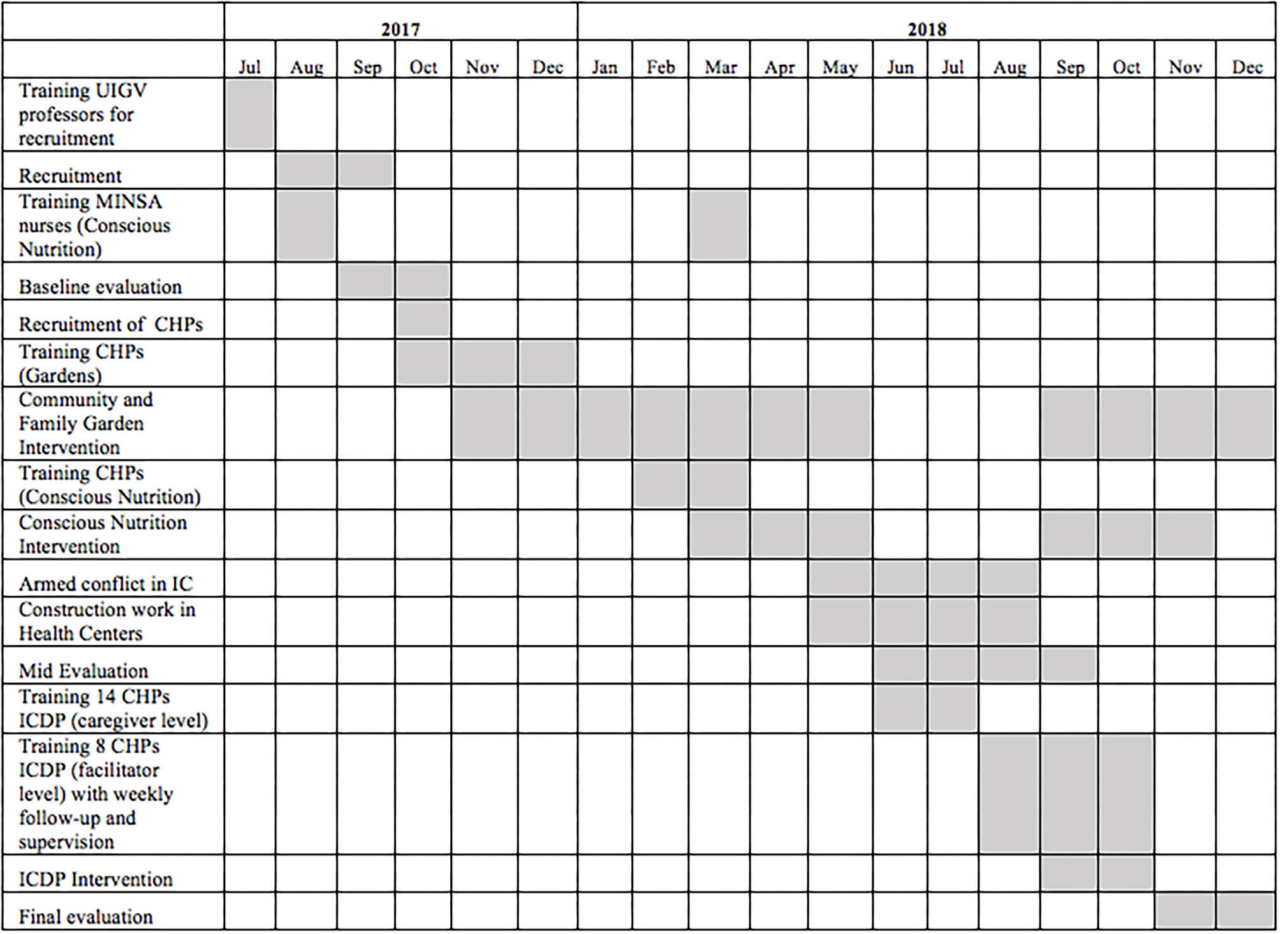
Timeline for Wawa Illari project.

This was a prospective, interventional, pre-post study that compared the occurrence of developmental delay in children aged 0–3 years between intervention and non-intervention communities in an urban settlement area in Lima, Peru. We investigated whether the combination of community/home gardens, workshops in conscious nutrition and international child development program (ICDP) methodology would positively affect the growth and development of children 0–3 against the background of ongoing current Peruvian Ministry of Health (MINSA) protocols in both control and intervention communities. The intervention consisted of three activities introduced in sequence (Figure 1): home gardens, nutrition workshops focused on awareness of child needs “conscious nutrition,” and “ICDP” workshops focused on caregiver-child interactions. Each of these interventions were intentionally adapted in response to challenges and feedback over the course of the study (Table 1). The children were monitored 3 times: at baseline, 8, and 12 months.

**Table 1:** Summary of adaptations to methodologies in response to specific field challenges.

**Initial plan**	**Field situation**	**Adaptations**
**Partnerships** • Experts in the methodology would train UIGV nursing school professors who, with their students, would recruit participants, implement and evaluate the interventions as part of the curriculum.	• After the initial training and recruitment, the UIGV realized that logistics, security, contractual and technical difficulties did not allow them to continue the partnership.	• Our “local team” in Lima took on the implementation, coordination and logistics for the entire intervention. • People from the intervention communities were hired and trained as CHPs to bring the methodology to families. • Nurses of the local Pachacámac Health Network supported the research component of the study.
**Garden intervention** • An agricultural engineer based in Lima was initially hired to lead planning and planting community gardens. • Community gardens would be planted in each small community on public land.	• Intervention families were scattered within CPRs over 5 km in a steep terrain making access to community gardens difficult • The agricultural engineer provided a program outline, budget and large donation of seeds from a local company, but the needed shift to a house-by-house methodology was not suitable for him. • There was land available only in 2 communities	• Two community gardens were established but the focus shifted to individual family gardens
• Families would pick-up from central locations seeds and small plants to start family gardens.	• Soil tests indicated no nitrogen in soil that was essentially ground rock, and families lacked transportation to obtain soil and other materials. • There was no rain in the region, the soil was arid and the area had a high solar radiation factor.	• A nurseryman hauled soil and compost for both community gardens. One of them required a fence, a protective mesh shade cloth and the installation of a water tank. • Fertile soil and recycled wooden produce boxes lined with porous fabric were provided seeds and seedlings and compost tea. • Motorcycle taxis were hired to help mothers get heavy and bulky elements home. The local team took materials to the homes of those who were absent.
• Training of volunteer CHPs in garden methodology over continuous sessions in one intensive week.	• The intensive training was insufficient given the varied level of education (elementary school to high school), with one local organic gardener, and given that the un- or under-employed CHPs (14 female and one male) needed to have some payment and also to take care of their homes and children.	• CHP's spent a full day at an organic educational farm run by retired agriculture professors to learn organic practices. • Learning was spread out over time, and refocused to transfer specific simple messages and tasks each week, through a learning system “gota-a-gota” (step-by-step). • The local organic gardener became our lead supervisor and educator for the on-going gardening workshops. • CHPs received a financial incentive.
**Conscious nutrition** • Community workshops delivered directly to caregivers at a community kitchens	• There was only one community kitchen in one of the intervention communities, and it lacked the minimal criteria of water and hygiene. • All mothers had difficulties meeting at specific times.	• CHP's received nutrition training from our team expert. • Given acceptability, feasibility and accountability of the “step-by-step” system, we continued applying it for the nutrition intervention. • CHPs were provided with one recipe/week, main ingredients and key messages about hygiene using printed material that was delivered directly to each family at their homes.
• Foods were to be purchased from a wholesale market, and distributed in small packages so CHPs could take them to the families	• The work load for wholesale purchase and preparation of individual packages was too high. • A cold-chain was not available and most of beneficiaries including CHPs did not have a refrigerator.	• One CHP had a small store in the community. The ‘local team’ coordinated with her the order and distribution of small packages for other CHPs and their respective families. Only non-perishable items were distributed.
**First post-intervention evaluation** • Evaluation of children 6 months after baseline, following the gardening and nutrition interventions	• There were several weeks of armed violence over land issues in one intervention community. CHPs expressed their own and their community's feelings of fear, uncertainty and abandonment by local institutions. We were concerned about the health risks for CHPs to visit families. • There was construction at the health clinics in Manchay and San Juan (IC) and Las Palmas' clinic (CC) was closed due to lack of water and energy supplies.	• Educational workshops were temporarily halted for the safety of the team. Monitoring of the situation was done using Whatsapp communication with CHPs. • The mid-evaluation was delayed by 2 months. Safety protocols were implemented. Trips and time spent in the field were restricted. • A stress questionnaire was developed for assessing the impact of conflict on children. • We provided a tent for evaluation of children in Manchay, and the San Juan health center also installed a tent. In las Palmas, the nurse made appointments to evaluate participants in a restricted schedule.
**ICDP methodology** • Training all 15 CHPs as facilitators of the ICDP methodology, and hiring them as part-time contract employees to cover health insurance.	• Seven CHPs declined the temporary part-time employee status because they did not want to lose the free government health insurance.	• Only 8 CHPs were hired as part-time employees. They received caregiver- and facilitator-level training and were remunerated based on the number of families they were able to visit. The other 7 CHPs received ICDP training but did not participate further.
• Intensive training in ICDP methodology over 3 days	• Even though we provided two levels of training, CHPs had difficulty recalling and transmitting the contents.	• We reinforced one ICDP guideline and one sensitization principle each week.
• ICDP workshops and supporting material are designed to train small groups of parents.	• The communities lacked appropriate locations for group gatherings and caregivers were unable to attend workshops at specific times. The challenge was to change the method of delivery but not the content.	• CHPs delivered the ICDP methodology directly to the homes of participants using the “step-by-step” learning system.

### Context

This study was conducted in urban settlements to the north and south of the district of Pachacámac, based on recommendation from nurses in the Pachacámac Public Health Center who identified these as the neediest communities in the region. This area, 59 kilometers south but within metropolitan Lima, features barren, rocky hills with scattered housing communities without paved roads. The valley floor has one main rutted dirt road, and multiple small farms known for strawberries. There were no large grocery stores or pharmacies, only small stores selling a limited range of foods. Most housing was precariously built of thin boards, and only one or two rooms. Some homes were made with bricks and plaster. There was no public water or sanitation system. Water was from wells stored in a large tank at the highest elevation in each community and delivered via plastic pipe to 50 gallon tanks at each family home. Community leaders were responsible for chlorinating the central tank water each month, but times and amounts of chlorine used were irregular. There was no public infrastructure for essential sanitation services or roads, though there were two public health clinics, two public preschools, one elementary school, one high school and several churches.

During 2018, the Ministry of Health (MINSA) conducted two campaigns to detect and treat anemia in both the control and intervention communities that overlapped with our nutrition and ICDP workshops. They also provided micronutrient supplements in the form of a sachet (1 dose): 12.5 mg of elemental iron, 5 mg of zinc, 160 μg of folic acid, 300 μg of vitamin A and 30 mg of vitamin C, to be administered daily for 1 year to children starting at 6 months of age (24).

### Community Selection, Sample Size Estimates, and Recruitment Procedures

The study area was divided as CPRs of North and South, which were separated by an urban area that served as a natural border and prevented contact between control and intervention communities. The southern region was inhabited by recent migrants from rural areas of Peru whereas the northern region was home to families that had been relocated from settlements during social programs. Selection of communities was based on inclusion within regions served by the Manchay and San-Juan (South), and Las-Palmas (North) health centers. Randomly, the southern CPRs were chosen as the intervention zone (Pampachica, Manchay Bajo, Lote B, Manchay Alto, Curva Zapata, and San Juan), and the northern CPRs were chosen as the control (Casitas, Rinconada and San-Miguel).

Sample size estimates were based on a prevalence of developmental delay of 14.6% in 0–3 year old children from low-middle income countries (7). Using OpenEpi software (25), we estimated that a sample size of 100 children in each of the control and intervention communities would be needed to detect with 95% confidence a decrease of 0.2 in the odds of developmental delay as defined by the Pan-American Health Organization (PAHO) (1).

Meetings were held with community leaders and families from the intervention and control areas to explain the project. Nursing professors of the Inca-Garcilaso-de-la-Vega University (UIGV) trained in technical and ethical recruitment procedures accompanied by their students and guided by MINSA nurses then visited homes with at least one child 0–3 years of age, to explain the study using picture cards. MINSA nurses also recruited children during regular growth and follow-up in the Manchay and Las Palmas health centers.

The two inclusion criteria were at least one child aged 0–3 years, and agreement of the caregiver to allow us to monitor the growth and development of their children in 3 evaluations distributed during the project duration and answer several questionnaires. In the case of the intervention communities, the caregivers also agreed to receive the proposed methodology, work in home gardens and attend training workshops. This was indicated by written informed consent. The exclusion criteria were chronic diseases or congenital malformations; no children had these conditions.

A total of 315 children were recruited, 157 from the control communities (CC) (71 boys and 86 girls), and 158 from the intervention communities (IC) (92 boys and 66 girls). At the end of the project, 127 children from the CC (58 boys, 69 girls) and 113 from the IC (65 boys, 48 girls) had completed at least two of three evaluations and were included for analysis.

### Intervention Methodologies

During the course of the project, a number of challenges emerged which required adaptation to our original design. These are summarized in Table 1.

#### “Step-By-Step” Learning System for Community Health Promoters (CHPs)

The research team identified 15 people of the IC who were willing to be trained as CHPs in each of the intervention methodologies and carry these messages to designated households. Training was adapted from intense sessions to a more “step-by-step” process with smaller and more focused specific training (ASM)^1^ that was more effective for transfer the knowledge to the families, regularly reinforced by experts. Throughout the training, the importance of encouraging parents to have a conversation with the children whether during gardening, cooking, washing hands, brushing teeth, or playing was highlighted. CHPs also learned to encourage caregivers to name things from plants to food ingredients and to stimulate the child's sensory engagement in each activity. This approach was designed to allow CHPs to develop technical skills and to improve self-esteem and build a trust relationship with families.

The local team had weekly meetings with CHPs where they provided feedback about their experiences, shared difficulties and findings, and learned from each other. Little by little, CHPs took “ownership” for each methodology and its contents. Materials were also provided at these weekly meetings.

Each CHP was responsible for 7–11 families and each family had a weekly lesson in gardening, and later in nutrition, cooking and the ICDP methodology.

#### Community and Home Gardens (On-Site Field Adaptations)

Two community gardens were created, one in Manchay, and one in San Juan. They served as sites for training for CHPs who also maintained the gardens. They were available to any family who wished to make use of the gardens.

In addition, individual family gardens were promoted. A model workshop was held for the CHPs where they were provided with two wooden slat boxes, one for leafy greens (24” × 18” × 8”) and one for root crops (24” × 18” × 11”), each lined with polyester porous agricultural feed bags. Vegetables were selected based on both on the nutritional content and cultural acceptance with a focus on high iron content foods: parsley, spinach, cabbage, swiss chard, carrots, green onions as well as turnips, beets, and radishes that have edible leaves as well as roots. CHPs planted their own boxes to take home and learned how to provide instruction when they visited families.

Boxes, soil, compost, seeds for most crops as well as parsley and green onion plants were provided to 107 families. The initial garden intervention was provided over 12 visits, and then continued throughout the project. A specific task was covered each week to ensure ongoing learning and a successful harvest: planting, organic fertilizers, pest management, crop harvesting and seed harvesting for garden sustainability. At each visit, the caregiver signed a register to confirm participation and receipt of materials and the CHPs took photos of each garden. The last community intervention was planting a lemon, orange, apple or peach fruit tree at every child's home in the IC, as a way to promote environmental health, soil improvement and a future fruit supply. Fruit trees were also planted at the Niña Maria community garden and San Juan public pre-school community garden and the San Juan health center for long term community benefits. At each visit, CHPs continued to supervise gardens and to distribute seeds when required.

#### Conscious Nutrition Intervention (On-Site Field Adaptations)

The second phase of intervention was designed to increase the likelihood that infants receive nutrients known to improve infant development. CHPs received 12 h of training in conscious nutrition and cooking from our nutrition advisor that emphasized the preparation easy and tasty recipes using affordable local foods rich in macro- and micro-nutrients known to be essential for child development. The lessons emphasized connecting with the colors, smells and tastes of each food, and the importance of the parent's feelings while preparing food for the child (26). CHPs were provided with one recipe/week, main ingredients and key messages about hygiene using printed material.

Each CHP delivered the supplies and lessons directly to each family at the family home after practicing in their own home. They stressed the importance of a diet with a variety of flavors, colors and smells; in eating using the five senses; making meals a happy experience for a loving interaction between caregivers and children; the use of traditional and new local recipes to increase the variety in the diet; and to incorporate the families' garden produce. Recipes also included the mixture of multiple micronutrient (MMN) powder with foods, following MINSA guidelines (24). Children responded with pleasure to all the recipes which encouraged mothers to put them into practice. The follow up of every family visit was also done by asking for participants' signatures and taking pictures of prepared foods.

#### Adaptation of International Child Development Program (ICDP)

The International Child Development Program (ICDP) (27), our third aspect of the intervention, has two levels of training. For the first ‘caregiver level,’ the local team provided 6 training sessions of 3 h/week whereby CHPs practiced the ICDP methodology with their own children in order to internalize the process. For the second ‘facilitator level,’ an additional 8, 2-h sessions focused on the 7 sensitization principles of the ICDP approach that promote “sensitization” rather than “teaching” in order to encourage caregivers to make positive changes. These sessions helped CHPs to facilitate parent-child interactions in a way that showed empathy toward parents and children through sharing of personal stories, putting themselves in the caregiver or child's place, demonstrations and role playing games, listening to caregivers and giving simple explanations. Through this process, CHPs understood the need to incorporate all the guidelines in each caregiver-child interaction given that they are mutually reinforcing. Eight CHPs participated in the facilitator training.

Eight ICDP workshops were provided at each home. They focused on the three ICDP forms of dialogue (emotional-expressive, meaning oriented and regulative dialogues) and covered by 8 specific ICDP guidelines (27, 28). The *emotional-expressive* form of dialogue comprises the first 4 guidelines: (1) show love and positive feelings to your child, (2) follow and adjust to your child's initiatives, (3) establish close communication, with or without words, and (4) praise and appreciate your child's efforts and achievements. The *meaning oriented* dialogue includes the next 3 guidelines: (5) establish shared focus and attention with your child, (6) provide meaning by naming and describing things and actions, and (7) expand on meaning by connecting, comparing and using creativity (songs, stories, painting etc.). The *regulative* dialogue contains the two parts of the last guideline: (8a) establish limits, norms and values, and (8b) guide your child's activity step by step toward a goal.

Caregivers learned to apply sensitization principles by sharing personal experiences, providing explanations and concrete examples, and facilitating role-games and direct interaction with children. Story books and didactic wooden toys were used. Though not part of the original ICDP material, they were helpful in promoting caregiver-child interactions during home visits.

### Monitoring of Children

Children were assigned to one of three health centers for monitoring based on proximity to the home: San-Juan or Manchay for IC, and Las Palmas for CC, and were followed by MINSA nurses in charge of the Growth and Development program who received specialized training for the research. The main outcome was the development of children, evaluated through the PAHO growth and development scale (29) which is also the basis for Peruvian guidelines for the follow-up of children under five (30).

#### Questionnaires

In addition to a baseline questionnaire administered only at the beginning of the study, questionnaires on child feeding, food security, and health of the child were administered at 0, 8, and 12 months, and the stress questionnaire was administered at 8 and 12 months.

##### Baseline

The baseline evaluation included questions on the caregiver and home, including age, education, marital status of the parents, number of people living in the household and in the same room with the child, presence of pets and vermin at home (31) In addtion, data on the following PAHO risk factors for developmental delay (1) were recorded: presence of maternal anemia, hypertension, urinary tract infection and other complications during the pregnancy of the participant child, weeks of gestation, infant birthweight, development of neonatal jaundice, exposure to wood or cigarette smoke at home, and frequent consumption of alcohol by someone in the household.

##### Child feeding

Caregivers were asked if the infant was currently receiving breastmilk (0: no, 1: yes). For those who were taking food, a locally adapted food frequency questionnaire was applied using a list of foods that were common in the region (32) (Supplementary Table 1). The nurse asked whether the child ate each item as part of their usual diet. Each item was given a score of one and variety of foods eaten by children was defined as the number of foods used to feed children in each evaluation period. Caregivers also indicated whether or not their child was receiving MMN supplements.

##### Food insecurity

Scores (0–3) were assigned to a positive response to the following questions that referred to the past 6 months for the household. Did you worry about having enough food due to lack of resources (0: no, 1: yes)? Was there a limitation in the types of foods that were consumed due to lack of resources (0: no, 2: yes)? Was there a lack of food at home due to lack of resources (0: no, 3: yes)? Was there a lack of appropriate foods for the child due to lack of resources (0: no, 3: yes)? The highest individual score was used to reflect the degree of food insecurity as follows: (0) no food insecurity, (1) mild food insecurity, (2) moderate food insecurity, and (3) severe food insecurity (33).

##### Health assessment

Nurses asked the caregiver about the presence and frequency of diarrhea (≥ 3 liquid stools/d) and respiratory infections (syndrome of cough and fever of any severity) following PAHO Integrated Management of Childhood Illness (IMCI) strategy (29), and number of visits to the doctor due to illness (including hospitalizations) in the last month. Children were considered to have persistent diarrhea or respiratory infections if the number of episodes recorded in the second and third evaluations were found to be equal or higher to that from the previous evaluation(s).

##### Stress

A novel stress assessment questionnaire was validated following a period of armed conflict that affected some CPRs of the intervention region at the end of conscious nutrition workshops. Nurses asked if, in the past 3 months, the caregiver or the family had lived in stressful situations. If the answer was “yes,” child stress was evaluated by asking caregivers if the child ate less than usual, slept less than usual, woke up scared, cried more frequently, looked sad, showed less desire to play, or was unable to play outside for safety reasons. A score of 1 was given to any affirmative answer, and the cumulative score (range 0–7) was calculated. For evaluating caregiver stress, nurses also asked if the stressful situation had affected caregiver's ability to get food for the child, if the caregiver easily got mad at the child, and if the caregiver was less willing to play with the child. Similarly, scores for positive answers ranged from 0 to 4.

##### Overall qualitative assessment of the intervention

At 8 and 12 months, caregivers were asked whether they incorporated their garden produce into the family meals, and whether they used nutrition and caregiver-child lessons and skills in daily life.

#### Anthropometry

Child weight, length/height and head circumference were measured three times (0, 8, and 12 months) following WHO standards (34). For infants, nurses used the Seca 354® digital scale for babies, the Seca 210® mobile measuring mat for baby and toddler, and the Seca 201® circumference measuring tape. For children 1–4 years, a Seca 769® Wireless Eye-Level Digital Scale with Height Rod was used. Z-scores of weight-for-age (WAZ), length-for-age (LAZ), weight-for-length (WLZ) and head-circumference-for-age (HCAZ) were calculated using the STATA 14 least mean squares method applied to WHO reference data (35).

#### Intestinal Parasites and Anemia

Stool samples, collected from children at the first and second evaluation periods, were examined for intestinal parasites using a sedimentation technique and for pinworms using the Graham's scotch tape test for pinworms. Heel or finger-prick blood samples were collected by nurses at the three evaluation periods and assayed hemoglobin (hemoglobinometer HemoCue Hb 301 Auto®). Anemia was defined as hemoglobin <11 g/dL (36).

#### Evaluation of Caregiver-Child Interaction

The ICDP program is usually evaluated with the help of pre- and post-intervention video (27, 28) which was not possible in our context due to technical constraints and ethical considerations. Instead, we used two approaches to evaluate caregiver-child interactions.

For an objective evaluation of ICDP workshops, we developed and validated a novel checklist based on gestures or expressions that reflected ICDP guidelines (27). MINSA nurses were trained in 3 sessions of 6 h to use this checklist when observing caregiver attitudes that reflected ICDP guidelines. In the last two evaluation periods, caregivers in both IC and CC were given the opportunity to interact with the child (diaper change, breastfeed, giving food and/or play) while the nurse, at a distance, observed the caregiver's application of the 8 ICDP guidelines (Supplementary Table 2). Nurses classified the interaction as (0) if the gesture/attitude was not present, (1) if observed occasionally, and (2) if observed frequently. A score adding nurse's observations was created for each guideline.

During the last visit to the families, a subjective evaluation of ICDP workshops was completed by IC caregivers. They used a Likert scale to provide a self-assessment of the application of the 8 guides. CHPs asked caregivers whether they would currently rate their attitudes about each ICDP guide as very low, low, medium, a lot, or very much. They asked the same question about their attitudes prior to the ICDP workshops. The CHPs provided a visual representation to highlight areas of improvement (Supplementary Figure 1).

#### Child Development Evaluation

Child development was evaluated using the PAHO standards based on the guidelines for Integrated Management of Childhood Illness (IMCI) (1). This instrument is widely used in Latin America to monitor child development as part of the routine health care. The instrument assesses reflexes, attitudes and skills based on age-specific milestones (< 2 months, ≥ 2 months and ≤ 2 years, > 2 and ≤ 6 years) (Supplementary Table 2) (29). Using PAHO guidelines, child development was classified into four general categories: “normal” if the child displayed all the reflexes/positions/skills corresponding to her or his age group and there were no risk factors; “normal development with risk factors” if the child displayed all the reflexes/positions/skills corresponding to her or his age group, but there were one or more risk factors; “developmental alert” if the child did not display one or more of the reflexes/positions/skills corresponding to the his or her age group; and “suspected developmental delay” if the child did not display one or more of the reflexes/positions/skills corresponding to the previous age group, or had a head circumference < 2SD or > 2SD, or presented three or more phenotypic alterations (29). In addition, the medical research team categorized PAHO milestones as indicators of motor, social/cognitive and language subgroups (Supplementary Table 2). Child's motor, social/cognitive and language development were considered as delayed if respective age-specific milestones were not observed across evaluations, or if milestones were absent in an evaluation when previously they were present.

### Statistical Analyses

Questionnaires completed by nurses were digitally transferred weekly. Database verification by a second digitizer was made at the end of each evaluation. STATA/IC 16.1 for Mac (StataCorp, TX, USA) was used for analyses.

At baseline, child, caregiver and household characteristics, as well as risk factors for developmental delay were compared between CC and IC using Chi^2^ or Fisher's exact test for frequencies, Kruskall-Wallis test for ages and Student's *t*-test for birth weights.

To determine differences in categorical variables across evaluation points separately for CC and IC, Cochran tests for equality of proportions in matched samples were performed. Chi^2^ or Fisher's exact test were used to compare CC and IC at each evaluation. The following categorical outcomes were evaluated: frequency of different degrees of food insecurity, presence of intestinal parasites, presence of anemia, presence of reported stress signs in children, proportion of children in each development category and proportion of children not achieving developmental milestones for motor, social/cognitive and language development.

Variables were compared across time-points using Kruskal-Wallis tests (episodes of diarrheal and respiratory infections, number of visits to the doctor due to illness in the last month, foods taken by children, child stress scores, and caregiver-child interaction scores), and one-way ANOVA (anthropometry Z-scores and hemoglobin, g/dL). To assess differences between IC and CC, Kruskal-Wallis and Student's *t*-test were used. In order to determine whether an outbreak of violence within the IC region influenced child stress, the participants from IC communities were subdivided into those with or without violence and compared with those in the CC communities. The equality of stress scores at 8 and 12 months and of caregiver application of ICDP guidelines were tested by using the Wilcoxon matched-pairs signed-rank test. Kruskal Wallis was used to assess difference between IC and CC at 8 and 12 months.

We explored associations of food insecurity, motor, social/cognitive or language delays as dependent variables using stepwise multiple logistic regression models. Our independent variables were: received the combined intervention (0: CC, 1: IC), gender (1: boy, 2: girl), age at final evaluation (months), number of pets, number of vermin in the home, maternal education (years), paternal education (years), number of people sharing the bedroom with the child, breastfeeding (0: no; 1: yes), number of episodes of diarrhea in the last month, number of episodes of respiratory infection in the last month, taking micronutrients (0: no, 1: yes), hemoglobin (g/dL) and child stress scale (0–7). The stepwise process took out variables with *p* > 0.15. Then, adjusted risk ratios were calculated using the post-estimation “adjrr” command in STATA, which adjusts for covariates and uses the Delta method to calculate confidence intervals (37). Missing data were not imputed and complete case analyses were performed. Final models were assessed for collinearity (variance inflation factor < 10) and stability of coefficients (condition number < 30).

Finally, to assess the specific impact of ICDP methodology on language development we ran logistic regression models for language delay and the score of caregivers' application of each ICDP guideline as observed by nurses. Risk ratios were adjusted for receiving the intervention.

### Ethical Aspects

The study received ethical approval from the National Institute of Child Health (registration OEAIDE-02997-2017) and from the Research Ethics Board at McGill University (REB File # 144-0817).

## Results

### Baseline Characteristics

Characteristics of children, their parents, their homes, as well as risk factors for developmental delay are presented (Table 2) for 127 children from the CC and 113 from the IC who had baseline data and data from at least one follow-up evaluation. Children of CC and IC did not differ by age or sex (Supplementary Figure 2), birthweight or age of the parents. A lower proportion of mothers in the IC breastfed their children. In both IC and CC, most women (84.4%) spent all day with their children, but most fathers (84.8%) spent ≤ 2 days/week. In the CC the mothers had more years of education but higher frequency of unstable unions than in IC; the CC also had fewer people sleeping in the same room as the child, fewer families with dogs or cats, but the presence of mosquitoes and rodents in the house was more frequent. Mothers of the IC reported hypertension during the pregnancy of the participating child more often than the mothers of the CC. Other PAHO risk factors for developmental delay did not differ between CC and IC (Table 2).

**Table 2:** Characteristics of children, caregivers and households at the baseline evaluation in control (*n* = 125–127) and intervention (*n* = 97–113) communities.

	**Control**	**Intervention**	***p*-value**
**Infant characteristics**			
Gender			
Boys	45.7%	57.5%	0.07
Girls	59.3%	42.5%	
Age (months), median (min-max)	14 (1–37)	14 (0–41)	0.76
**Breastfeeding^a^**			
Exclusive	81.1%	67.5%	**0.028**
Mixed	17.3%	24.7%	
None	1.6%	7.8%	
Birthweight (g), mean ± SD	3,369 ± 453	3,322 ± 543	0.24
**Parent characteristics**			
**Maternal marital status**			
Single mother	0.0%	3.7%	**0.001**
Unstable union	8.7%	0.9%	
Stable union	91.3%	95.4%	
Mother's age (years), medium (min-max)	29 (17–46)	29 (16–50)	0.20
Mother finished secondary school, %	76.3%	61.5%	**0.013**
Father's age (years), median (min-max)	32 (18–58)	32 (19–67)	0.74
Father finished secondary school	88.0%	76.0%	**0.017**
**Household characteristics**			
> 5 people in household	29.1%	25.5%	0.53
≥ 3 people in same bedroom with child	12.7%	48.6%	**< 0.0001**
**Pets**			
Dog	37.8%	58.7%	**0.001**
Cat	26.0%	39.4%	**0.027**
Other	2.3%	1.8%	0.57
**Vermin**			
Flies	62.2%	73.4%	0.07
Mosquitoes	63.8%	42.2%	**0.001**
Cockroaches	38.6%	27.5%	0.07
Rodents	44.9%	29.4%	**0.014**
**Food insecurity**			
None	32.3%	44.9%	**< 0.0001**
Mild^b^	2.4%	5.6%	
Moderate^c^	60.6%	14.9%	
Severe^d^	4.7%	34.6%	
**PAHO's known risk factors for developmental delay**			
Anemia in pregnancy	29.1%	38.1%	0.15
Hypertension in pregnancy	8.7%	20.6%	**0.010**
Urinary tract infection in pregnancy	57.5%	52.6%	0.46
Other complications of pregnancy	13.4%	17.4%	0.39
Preterm birth (< 37 weeks)	2.4%	6.4%	0.12
Low birthweight (< 2,500 g)	3.2%	7.5%	0.12
Neonatal jaundice	19.8%	27.1%	0.19
Exposure to smoke (firewood or cigarette)	16.5%	25%	0.11
Presence of someone with alcohol problems at home^e^	39.4%	28.2%	0.07

a *n = 77 in control community*.

b *Mild food insecurity: the caregiver is worried about not having enough money to buy food*.

c *Moderate food insecurity: Food quality is compromised due to lack of resources*.

d *Severe food insecurity: Lack of food at home or not sufficient food for the child due to lack of resources*.

e *Alcohol problems refer to frequent, non-occasional intake of alcohol, or heavy intake of alcohol until drunkenness. Bold numbers indicate significant differences at p < 0.05*.

### Impact of the Interventions on Nutrition and Health

#### Food Insecurity and Variety of Foods Eaten by Children

**Figure 2:**
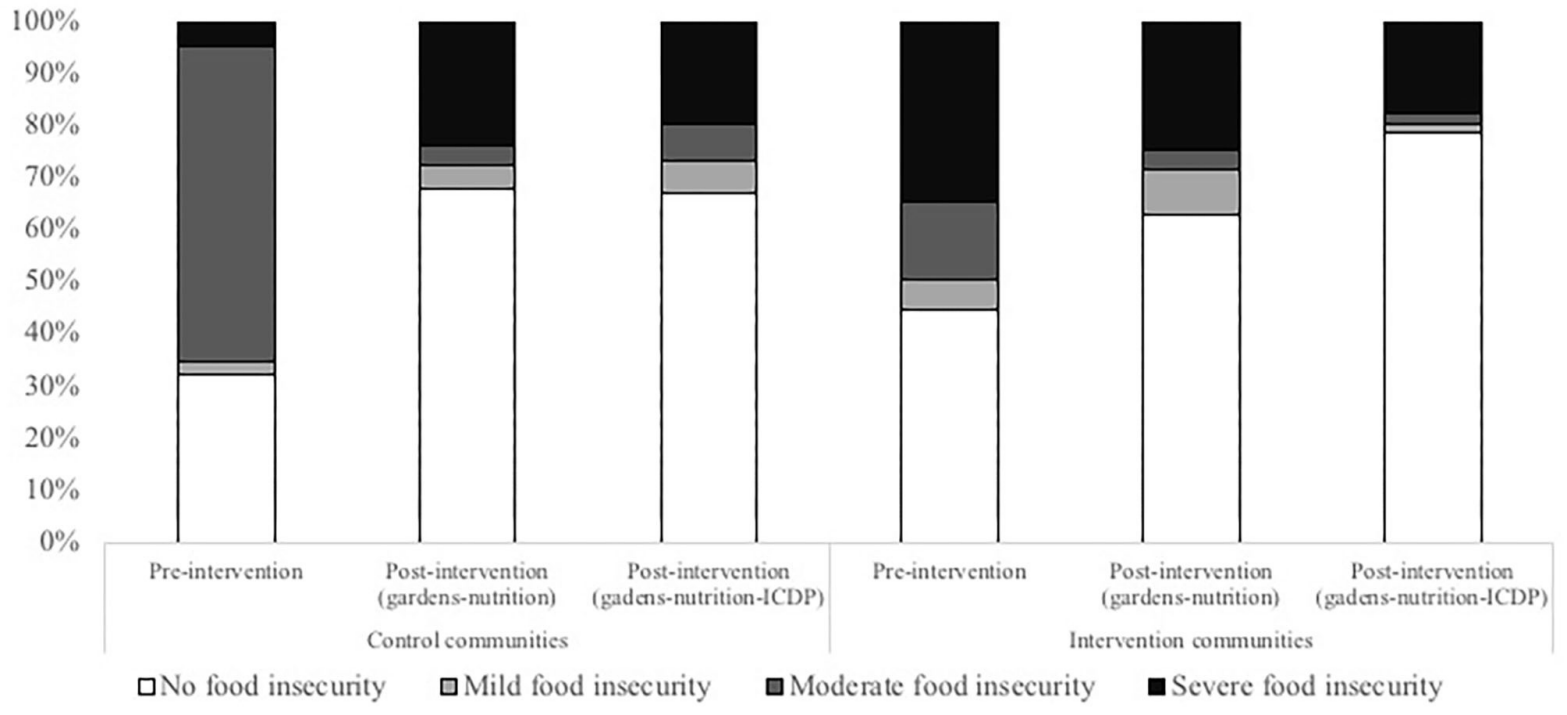
Percentages of food insecurity (no food insecurity, mild, moderate and severe food insecurity) at three time-points in control and intervention communities.

At baseline, moderate food insecurity was more frequent in the CC, but the IC had a higher percentage of severe food insecurity. The level of food insecurity did not differ between CC and IC at 8 or 12 months. However, within the IC, food security improved with a higher percentage of households reporting no food insecurity in the IC compared with the CC in the last evaluation (IC: 75.2%, CC: 59.1%, *p* = 0.017) and a decrease in severe food security over time (32.7–16.8%, *p* < 0.0001) (Figure 2).

At baseline, children in CC ate a greater variety of foods compared with IC (average of 21 vs. 14 items, respectively, *p* < 0.001). Although the difference remained across evaluations, the diversity of foods consumed by children increased more than 2-fold over the study in both CC and IC (Supplementary Figure 3).

#### Reported Frequency of Visits to the Doctor Due to Illness, Diarrhea and Respiratory Infection Episodes in the Last Month

During the pre-intervention evaluation, caregivers of children in the IC reported a higher frequency of visits to the doctor due to illness (Supplementary Figure 4A), diarrheal (Supplementary Figure 4B) and respiratory disease (Supplementary Figure 4C) in the last month when compared to the CC (all *p* = 0.0001). The number of visits to the doctor and episodes of diarrhea and respiratory infections in the IC declined at 8 and 12 months (*p* = 0.0001).

#### Intake of Multiple Micronutrients, Hemoglobin Concentrations and Presence of Anemia

Mothers in the CC reported giving MINSA-delivered MMN powder to children more often than in the IC. The number of children in the IC receiving MMN powder significantly increased during the course of the project (Table 3) but did not reach the proportion of the CC.

**Table 3:** Health characteristics of children from control communities (CC) and intervention communities (IC) at baseline, at 8 months post-intervention (gardens-nutrition), and at 12 month post-intervention (gardens-nutrition-ICDP)^1^^,^^2^.

		**Baseline**	**Post-intervention (gardens-nutrition)**	**Post-intervention (gardens-nutrition-ICDP)**	***p*-value**
**Clinical Indicators**					
Micronutrient intake	CC	63.0%	70.9%	71.2%	0.068
	IC	28.7%	58.2%	47.2%	**0.0001**
		***< 0.0001***	*0.061*	***< 0.0001***	
Hemoglobin (g/dL), mean ± SD	CC	11.0 ± 0.9^b^	11.6 ± 0.9^aA^	11.6 ± 1.0^aA^	< 0.0001
	IC	10.9 ± 1.2^b^	11.3 ± 0.9^aB^	11.3 ± 1.1^aB^	**0.010**
		*0.208*	***0.044***	***0.027***	
Anemia	CC	44.9%	20.0%	25.9%	**< 0.0001**
	IC	52.3%	35.2%	27.4%	***0.009***
		*0.256*	***0.016***	*0.807*	
Anthropometry^3^					
WAZ, mean ± SD	CC	0.45 ± 0.9	0.35 ± 1.0	0.36 ± 0.9	0.697
	IC	0.39 ± 1.0	0.21 ± 1.0	0.18 ± 1.0	0.278
		*0.322*	*0.148*	*0.093*	
LAZ, mean ± SD	CC	−0.32 ± 1.3	−0.37 ± 1.0	−0.38 ± 1.0	0.893
	IC	−0.43 ± 1.1	−0.71 ± 1.1	−0.69 ± 1.1	0.123
		*0.210*	***0.009***	***0.013***	
WLZ, mean ± SD	CC	0.87 ± 0.9	0.81 ± 1.1	0.87 ± 1.0	0.887
	IC	0.88 ± 1.1	0.86 ± 1.0	0.86 ± 1.1	0.991
		*0.472*	*0.374*	*0.497*	
HCAZ, mean ± SD	CC	0.37 ± 1.1	0.22 ± 1.1	0.42 ± 1.1	0.377
	IC	0.29 ± 1.2	0.39 ± 1.2	0.54 ± 1.2	0.318
		*0.294*	*0.145*	*0.219*	
**Developmental Scale**					
Normal development^4^	CC	15.7%	16.4%	19.6%	0.174
	IC	11.4%	15.6%	12.9%	0.135
		*0.342*	*0.877*	*0.181*	
Normal development	CC	52.0%	50.9%	45.5%	0.283
with risk factors^5^	IC	65.7%	60.0%	59.3%	0.423
		***0.035***	*0.199*	***0.042***	
Developmental alert^6^	CC	15.7%	20.0%	20.5%	0.161
	IC	12.4%	8.9%	11.1%	0.260
		*0.465*	***0.029***	*0.056*	
Suspected developmental delay^7^	CC	2.4%	0.9%	2.7%	0.368
	IC	2.9%	3.3%	2.8%	0.882
		*0.565*	*0.239*	*0.641*	
**Not Achieving Milestones for Age**					
Motor delay	CC	8.7%	9.8%	4.5%	0.230
	IC	9.4%	7.8%	6.5%	0.465
		*0.838*	*0.612*	*0.510*	
Social/cognitive delay	CC	7.9%	4.5%	3.6%	0.761
	IC	6.6%	5.6%	8.3%	0.627
		*0.710*	*0.722*	*0.113*	
Language delay	CC	18.9%	22.3%	21.4%	0.627
	IC	9.4%	10.0%	9.3%	0.664
		***0.042***	***0.020***	***0.013***	

1 *Sample sizes: Baseline: CC = 127; IC = 105–112; 8 months: CC = 110; IC = 90–93; 12 months: CC = 102–112; IC = 106–108*.

2 *Percentages were compared by Chi^2^ or Fisher's exact test. Means were compared across time by one-way ANOVA over three times or between IC and CC by Student's T-test. Different lower case superscripts represent significant differences over time; different upper case superscripts represent differences between CC and IC*.

3 *WAZ, Weight for age Z-scores; LAZ, length for age Z-scores; WLZ, weight for length Z-scores; HCAZ, head circumference for age Z-scores*.

4 *Normal development: child displays all reflexes/positions/skills corresponding to age group and no risk factors*.

5 *Normal development with risk factors: child displays all reflexes/positions/skills corresponding to age group, but one or more risk factors*.

6 *Developmental alert: child does not display one or more reflexes/positions/skills corresponding to age group*.

7 *Suspected developmental delay: child does not display one or more reflexes/positions/skills corresponding to previous age group, or has HCAZ < 2SD or > 2SD, or has three or more phenotypic alterations. Bold p values indicate significant difference at p < 0.05, and Italics denote comparisons between Control and Intervention Communities during the same evaluation point*.

Although children in the CC had higher hemoglobin concentrations than those from IC in post-intervention assessments, we observed an increase in hemoglobin concentrations and decreased percentages of anemia in both IC and CC throughout evaluations (Table 3).

The prevalence of nematode infections (*Enterobius vermicularis, Strongyloides stercoralis* and *Hymenolepis nana)* was very low, but higher at baseline in the IC than CC (14.7 vs. 4.7% respectively, *p* = 0.022). On the other hand, over 30% of children had one or more protozoan parasite, most commonly *Giardia lamblia*. Other protozoa found in the population included *Blastocystis hominis, Chilomastix mesnili, Endolimax nana, Cryptosporidium parvum, Iodamoeba butschlii* and *Entamoeba coli*. Even though diagnosis and referral for treatment of intestinal parasites occurred through MINSA in both control and intervention communities, the prevalence of *Giardia* at the 8 month evaluation had increased in the intervention community (4.4 to 25.6%, *p* < 0.0001).

#### Anthropometry

**Figure 3:**
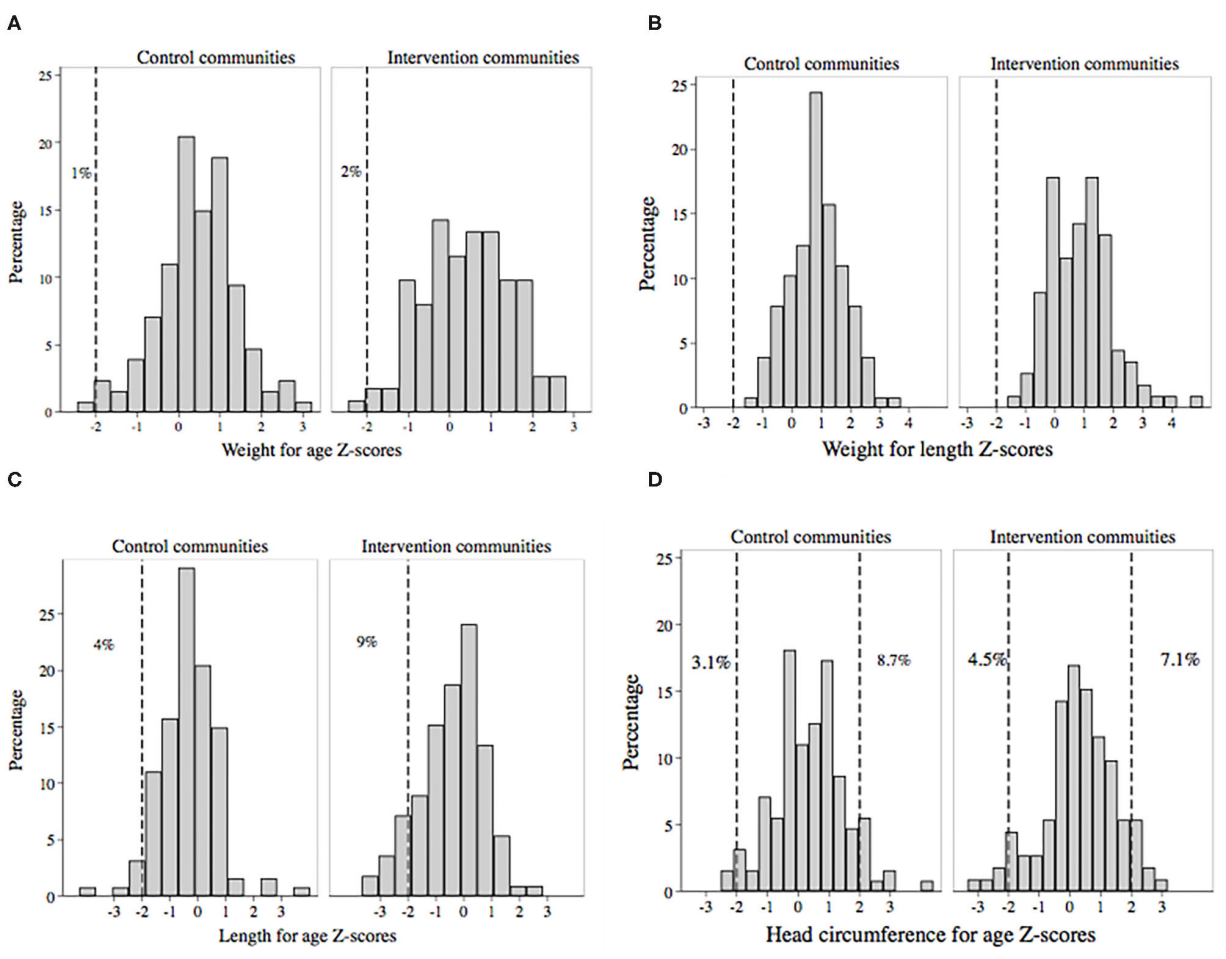
Histograms of anthropometric measurements (Z-scores) in children 0–3 years from the control and intervention communities: **(A)** weight for age, **(B)** weight for length, **(C)** length for age, **(D)** head circumference for age at baseline evaluation.

In general, children from CC and IC were within the normal range for weight, with very low prevalence of WAZ < −2 SD (Figure 3A) and no children with WLZ < −2 SD (Figure 3B). LAZ < −2 SD was found in 4% of CC children and 9% of IC children at baseline (Figure 3C), and at 8 and 12 months the LAZ was lower in IC than CC children (Table 3). Abnormal head circumferences HCAZ (< −2 or > 2 SD) were present in 11.8% of children from the CC, and in 11.6% of children from the IC (Figure 3D).

### Impact of Interventions on Child Development

When classifying children according to PAHO developmental scale, a higher proportion of children from the IC were categorized as “normal development with risk factors” at baseline and at 12 months (Table 3). At 8 months, after the intervention with gardens and nutrition but before the ICDP intervention began, the proportion classified as “developmental alert” was lower in the IC compared with the CC. There was no significant difference in the proportion of any of the developmental groups within communities over time.

When comparing achievement of developmental milestones, language delay for age was observed less frequently in children from the IC than CC at all time-points, but the percentage of children with language delay did not significantly differ over time. No differences were observed across evaluation periods or between CC and IC with regard to adequate motor development and social/cognitive development.

#### Child and Caregiver Stress

Due to the wave of violence in some of our intervention communities, we compared the presence and scores of stress in children and caregivers across evaluations and among participants in IC with and without violence and in CC communities. At 8 months, higher child and caregiver stress scores (*p* = 0.030 and 0.001, respectively) were observed in the IC communities with violence compared with non-affected communities. At 12 months, both child and caregiver stress scores were lower in the affected community (*p* = 0.026 and 0.049, respectively) than at 8 months. Unexpectedly, at 12 months, both child and caregiver stress were highest in the CC (*p* = 0.028 and 0.003, respectively).

#### Caregiver-Child Interaction

The subjective caregiver evaluation of their own application of ICDP guides showed that all IC parents felt they had improved their skills and qualities as caregivers.

The objective assessment by nurses is shown in Table 4. Previous to the ICDP intervention, CC caregivers were better at following the child's lead (Guideline 2), verbal and non-verbal communication (Guideline 3), and helping the child to focus his/her attention and share experiences (Guideline 5), whereas IC caregivers were better at praising what the child manages to do (Guideline 4), helping the child to make sense of his/her world (Guideline 6), widen his/her experience (Guideline 7), and to learn rules, limits and values (Guideline 8a). After the ICDP intervention, caregivers in both IC and CC showed improvement in all guidelines, but caregivers receiving the ICDP intervention had higher scores than CC caregivers in Guidelines 1 (showing love, *p* = 0.015), 4, (*p* = 0.0001), 6, (*p* = 0.0001), 7 (*p* = 0.0001), 8a (*p* = 0.0001) and 8b (accompanying the child to learn step by step, *p* = 0.012). At 12 months, IC caregivers had reached the level of CC caregivers in those guidelines that had been lower at 8 months.

**Table 4:** Scores reflecting caregiver interactions with their child^a^, based on observations by nurses of specific behaviors for each ICDP guideline^b^. Scores were compared between 8 and 12 months using the Wilcoxon matched-pairs signed-rank test, and between intervention communities (IC) and control communities (CC) using Kruskal Wallis test.

	**Community**	**Post-intervention (gardens-nutrition) at 8 months**	**Post-intervention (gardens-nutrition-ICDP) at 12 months**	***p***
Guideline 1	CC	2.8 ± 1.3	4.8 ± 1.6	< 0.0001
	IC	2.5 ± 2.2	5.4 ± 2.3	< 0.0001
	*p*	*0.131*	***0.015***	
Guideline 2	CC	2.3 ± 1.1	3.4 ± 1.2	< 0.0001
	IC	1.6 ± 1.5	3.6 ± 1.9	< 0.0001
	*p*	*0.0001*	*0.788*	
Guideline 3	CC	4.1 ± 1.6	6.3 ± 2.2	< 0.0001
	IC	3.4 ± 2.8	6.5 ± 3.2	< 0.0001
	*p*	*0.0008*	*0.383*	
Guideline 4	CC	0.7 ± 0.9	1.8 ± 0.9	< 0.0001
	IC	1.2 ± 1.2	2.5 ± 1.3	< 0.0001
	*p*	*0.011*	***0.0001***	
Guideline 5	CC	1.3 ± 0.9	2.1 ± 0.8	< 0.0001
	IC	0.8 ± 1.0	2.1 ± 1.4	< 0.0001
	*p*	*0.0002*	*0.671*	
Guideline 6	CC	0.6 ± 0.6	1.5 ± 1.2	< 0.0001
	IC	1.4 ± 1.6	3.1 ± 2.0	< 0.0001
	*p*	*0.0005*	***0.0001***	
Guideline 7	CC	0.2 ± 0.7	1.7 ± 1.6	< 0.0001
	IC	1.3 ± 1.9	3.6 ± 2.6	< 0.0001
	*p*	*0.0001*	***0.0001***	
Guideline 8a	CC	0.4 ± 0.9	2.2 ± 1.2	< 0.0001
	IC	1.3 ± 1.3	3.2 ± 2.0	< 0.0001
	*p*	*0.0001*	***0.0001***	
Guideline 8b	CC	0.9 ± 1.5	3.5 ± 1.7	< 0.0001
	IC	1.3 ± 1.6	4.3 ± 2.6	< 0.0001
	*p*	**0.060**	***0.012***	

a *For each behavior associated with a single guideline (see Table 7), nurses classified the caregiver-child interaction as (0) if the gesture/attitude was not present, (1) if observed occasionally, and (2) if observed frequently. A score summing the values for each behavior was then created for each guideline*.

b *Guideline 1: Showing love*.

*Guideline 2: Following child's lead*.

*Guideline 3: Verbal and non-verbal communication*.

*Guideline 4: Praising and appreciating what the child manages to do*.

*Guideline 5: Helping the child to focus his attention and share his experiences*.

*Guideline 6: Helping the child to make sense of his world*.

*Guideline 7: Helping the child to widen his experience*.

*Guideline 8a: Helping the child to learn rules, limits and values*.

*Guideline 8b: Accompanies the child to learn step by step*.

#### Multiple Regression Analyses for the Persistence or Worsening of Food Insecurity

A multiple logistic regression analysis showed that after controlling for multiple confounders, receiving the intervention reduced the risk of worsened/persistent food insecurity at 12 months. On the other hand, a higher child stress score and more pets in the home increased the risk of worsened/persistent food insecurity (Table 5).

**Table 5:** Multiple logistic regression analysis for worsening or persistence of food insecurity at the end of the project.

**Worsening or persistence of food insecurity**	**ARR ± SE**	**95% CI**	***p*-value**	**Model**
Received interventions	0.20 ± 0.09	0.08, 0.51	0.0001	*n* =199
Child stress score	1.64 ± 0.20	1.29, 2.09	< 0.0001	*p* < 0.0001
Number of pets in the household	1.73 ± 0.38	1.13, 2.66	0.004	Pseudo *R^2^* = 0.199
Maternal education, years	0.91 ± 0.01	0.89, 0.93	0.017	VIF = 1.05 Condition number: 10.45

*Adjusted risk ratios (ARR) are reported*.

#### Multiple Regression Analyses for Motor, Social/Cognitive, and Language Delay

Of the possible parental, environmental, biological and stress factors that could have affected child development, only a few variables were significantly associated with developmental indicators.

**Figure 4:**
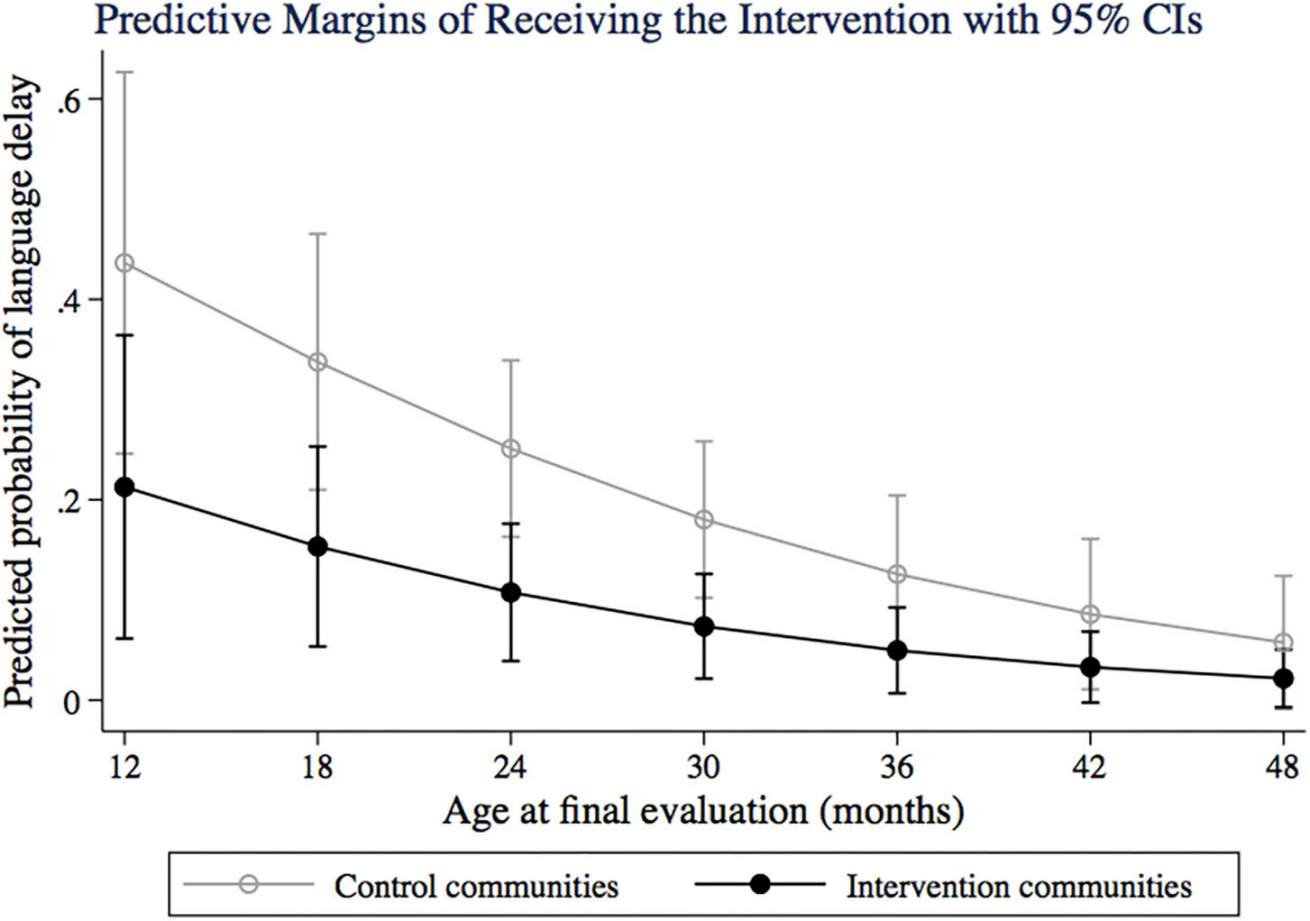
Predictive probability of language delay for control and intervention communities, at different age groups (12–50 months). Predictive margins are adjusted by number of diarrheic episodes in the last month.

Receiving the intervention package was not associated with an altered risk of motor or social/cognitive delay, but receiving MMN (which was included in recipes during nutritional workshops) decreased the risk of motor delay (Table 6A). A higher number of pets at home was associated with increased risk of both motor (Table 6A) and social/cognitive delay (Table 6B), and children having a higher stress score had a trend to a higher risk of motor and social/cognitive delay. Receiving the intervention was associated with 60% decreased risk of language delay (Table 6C) and children with higher number of diarrheal episodes had a borderline increased risk of language delay (Table 6C). The predicted probability of language delay when receiving or not the intervention at different age groups is shown in Figure 4.

**Table 6:** Multiple logistic regression analysis for (A) motor delay, (B) social/cognitive delay and (C) language delay at the end of the project.

**A: Motor delay**	**ARR ± SE**	**95% CI**	***p*-value**	**Model**
Received interventions	0.76 ± 0.41	0.26, 2.22	0.619	*n* =197 *p* = 0.0008
Micronutrient intake	0.12 ± 0.09	0.03, 0.56	0.004	Pseudo *R^2^* = 0.225
Child stress score	1.36 ± 0.24	0.95, 1.93	0.097	VIF = 1.07
Pets in the home (number)	3.24 ± 1.31	1.47, 7.14	0.0005	Condition number: 4.65
**B: Social/cognitive delay**	**ARR** **±** **SE**	**95% CI**	***p*****-value**	**Model**
Received interventions	1.71 ± 0.99	0.54, 5.35	0.342	*n* = 197 *p* = 0.0031
Micronutrient intake	0.39 ± 0.23	0.13, 1.24	0.108	Pseudo *R^2^* = 0.198
# pets in the home	2.72 ± 0.99	1.33, 5.55	0.0004	VIF = 1.08
Child's stress scale	1.33 ± 0.21	0.97, 1.83	0.082	Condition number: 11.88
Maternal education (years)	1.28 ± 0.18	0.96, 1.69	0.300	
**C: Language delay**	**ARR** **±** **SE**	**95% CI**	***p*****-value**	**Model**
Received interventions	0.39 ± 0.15	0.19, 0.82	0.006	*n*=197 *p* = 0.0004
Age (months)	0.97 ± 0.004	0.96,0.98	0.009	Pseudo *R^2^* = 0.107
Diarrheal episodes in the last month (number)	1.77 ± 0.49	1.03, 3.04	0.064	VIF = 1.01 Condition number: 7.50

*Adjusted risk ratios (ARR) are reported^a^*.

a *For each dependent variable, the analysis was based on whether the developmental delay was detected at 12 months regardless of status at baseline*.

In order to further evaluate the impact of ICDP methodology on language development, we ran multiple logistic regression models for language delay and the application of each ICDP guideline at 12 months, controlling for receiving the intervention. We found that the application of Guideline 8a (firmly correcting the child providing options and explanations), and 8b (accompanying the child step by step when trying something new) significantly decreased the risk of language delay (Table 7).

**Table 7:** Logistic regression models for the risk of language delay and the cumulative score for all attitudes associated with each guideline as observed by nurses watching caregivers interacting with their child, applying ICDP guidelines controlling for type of community (control vs. intervention).

**Language delay**	**ARR ± SE**	**95% CI**	***p*-value**
Showing love (Guideline 1, score)	0.93 ± 0.07	0.81, 1.07	0.468
Following child's lead (Guideline 2, score)	0.88 ± 0.08	0.74, 1.05	0.315
Verbal and non-verbal communication (Guideline 3, score)	0.99 ± 0.06	0.88, 1.2	0.936
Praising and appreciating what the child manages to do (Guideline 4, score)	0.80 ± 0.10	0.62, 1.03	0.197
Helping the child to focus his attention and share his experiences (Guideline 5, score)	0.79 ± 0.10	0.62, 1.01	0.167
Helping the child to make sense of his world (Guideline 6, score)	0.90 ± 0.09	0.74, 1.10	0.375
Helping the child to widen his experience (Guideline 7, score)	0.90 ± 0.07	0.77, 1.05	0.273
Helping the child to learn rules, limits and values (Guideline 8a, score)	0.73 ± 0.06	0.62, 0.85	**0.010**
Accompanies the child to learn step by step (Guideline 8b, score)	0.81 ± 0.04	0.73, 0.91	**0.024**

*Adjusted risk ratios (ARR) ± standard errors (SE) and 95% confidence intervals are reported*.

*Gestures/behavior observed to evaluate the presence of ICDP guides*.

*Guideline 1: the caregiver caresses, kisses, hugs, comforts the child*.

*Guideline 2: demonstrates interest in what the child does, is sensitive to child's intentions, plays what the child proposes*.

*Guideline 3: speaks to the child, smiles at him, looks at him in the eye, communicates at child's level of understanding, uses empathic tone of voice*.

*Guideline 4: praises what the child does well, encourages him*.

*Guideline 5: attracts his attention, joins child's interests*.

*Guideline 6: names objects, colors, counts numbers*.

*Guideline 7: gives explanations about the surroundings, makes comparisons, sings songs, tells stories*.

*Guideline 8a: when correcting the child, the caregiver presents options, provides explanations and is firm*.

*Guideline 8b: when the child tries to do something new, the caregivers accompanies the child in what he does, explains step by step, lets him try his best, provides support without interfering and accompanies child to achieve child's goal. Bold numbers indicate significant differences at p < 0.05*.

## Discussion

Children from deprived urban settlements are typically at higher risk of developmental delay, lower achievement, and more behavioral and emotional problems than children living in more favored contexts (38, 39), and interventions that normally work in other communities are not as useful for children from slums, as observed for nutritional programs (40). This study of a semi-urbanized population living in conditions of poverty with limited access to water-sanitation and other government services identified multiple risk factors that may preclude children from fully achieving their potential from among environmental, infectious and poverty-related stressors. Our interventions were led by CHPs selected from among parents within our IC who learned and transmitted the methodology to other caregivers using a “step-by-step” learning system. Both subjective and objective evaluation revealed that caregivers and their children had benefitted from the training as our focus on community/home gardens, workshops on conscious nutrition and promotion of parenting skills using ICDP methodology positively impacted child development. At baseline, food insecurity, diarrhea and respiratory infections were common but all were lowered following the intervention. The benefit on food insecurity persisted after controlling for multiple confounders. We also observed, at baseline, that 16.4% of children in the study had not achieved one or more of the age-specific developmental milestones. Following our combined interventions, this percentage declined. Furthermore, receiving the interventions reduced the risk of language delay. Two factors not linked to the intervention also influenced the risk of motor and social/cognitive delay: the intake of MMN decreased the risk of motor delay but having more pets increased it. More pets was also a risk factor for social/cognitive delay. Our proof of concept showed that a multi-sectorial approach can improve early child development in marginalized communities.

### Community-Based Participatory Research: The Impact on CHPs and Caregivers

The fundamentals of participatory research were critical to the implementation of this multi-sectorial study. The bidirectional communication between high- and low/medium-income countries through our project ensured that our research was grounded in social justice (41). Consistent with participatory research, our project focused on the mutual learning relationship between the international and local project team, the nurses, and the CHPs. This allowed the methodology to reach the most vulnerable families and provided us with valuable information on both obvious and hidden needs of these communities. Our work adds to evidence suggesting that interventions that promote nurturing care can be included as part of basic community and public health interventions (4). A review of community-based interventions for optimizing early childhood in low resource settings highlighted the importance of caregiver-child interactions (42). We confirmed this and expanded it to include gardens and nutritional components.

In order to go beyond cultural elements to reach individuals in targeted communities, interventions were modified to fit capacities of the project team and community settings (41). In addition, two new evaluation tools were developed. Our child stress instrument allowed us to identify and quantify stress in young children, to observe that stress was associated not only with an episode of violence but also with other unidentified causes, and to detect the association of child stress with food insecurity. Furthermore, borderline associations of child stress with motor and social/cognitive developmental delay show the need to consider child stress in future studies. Our novel checklist for evaluating caregiver-child interactions allowed us to objectively evaluate the ICDP methodology given that video recording used in previous studies (28) was not possible. This revealed the improvement of parenting skills in our IC but also in the CC.

One benefit of the project was the empowerment, enhanced communication skills and increase in self-confidence of the CHPs, gained through workshops with project experts and reinforced in their weekly visits to their families. This not only benefitted caregivers but also transformed the lives of the CHPs. At the end of the project, one CHP was hired by the community public health center to be a public health worker. Two had the courage to end an abusive partner relationship, and another started her own door-to-door egg business as a result of the confidence she gained in approaching people through her training and weekly educational visits to families. One CHP with a handicapped child agreed to do a presentation on ICDP to a regional gathering of parents of handicapped children because the program directors had heard her present to her own local support group. One was pregnant during the project and was able to apply all the methodology at home with her new baby. Families turned to them as resources for health and parenting information and were recognized by local authorities for their capacities. In parallel with their empowerment comes a responsibility to financially remunerate the time and efforts of CHPs that were the foundation for building community capacity. The multiple health risks that CHPs face in reaching remote households and during the wave of community violence in daily activities should be balanced with institutional support and CHPs should not be expected to perform their work on a voluntary basis. As has been noted by many others, the enhanced skills and abilities of CHPs need to be formally recognized financially (43, 44). The sustained caregiver engagement over the duration of the project suggests that the project was sustainable.

### Food Insecurity as a Target of the Interventions

Food insecurity is a known determinant of inadequate child growth (45, 46), and a reduction in food insecurity was an important achievement of the project, probably attributable to the creation of home gardens. There is growing evidence that home gardens have a positive impact on children's diet diversity, anthropometry (11), infectious diseases (47) and anemia (48). Although, home gardens were not associated with food security in a study from the Philippines (49), in the present study we showed a significant association of the combined intervention including gardens, on food security. Our study supports the fact that simple interventions could mitigate the impact of the conditions of extreme poverty and adverse circumstances on child development.

### Language Development

Interventions aimed at increasing children's stimulation can have a significant impact on language development (50). Although ICDP methodology was key for achieving the improvement of early child language development, besides its proven effect on parental skills (51, 52) and on decreasing difficulties in older children (52), ours is the first study to provide evidence that the ICDP methodology reduces the risk of language delay in children under five. We attribute this to the emphasis on treating the child as a person, giving the child a role in everyday family life, and explaining to the child what exists and happens in the environment (28). Among ICDP guidelines, the two belonging to the regulative dialogue (8a, 8b) emerged as determinants in reducing the risk of language delay. Those guidelines involve clear and articulate communication with explanations of why and consequences. Furthermore, ICDP guidelines were applied to concrete day-to-day activities such as meals and gardening times. Giving quality food, with a feeling of love, as well as the participation of children (when they were old enough) in the planting and preparation of food was also reinforced throughout the project.

Broader factors are also involved in language development. A recent meta-analysis of risk factors for developmental delay in low-medium income countries identified that lower maternal education was associated with lower child cognitive scores, that lack of access to clean water and sanitation was associated with lower cognitive and motor development, and that lack of clean water was associated with lower language scores but that diarrhea was not significantly associated with development (53). Interestingly, our nutrition and garden workshops were associated with a reduction in episodes of both diarrhea and respiratory infections, and furthermore the number of diarrheal episodes in the month prior to our final evaluation had a borderline impact on increasing the risk of language delay. This suggests that the reduction in language delay may be associated with improvements in child health due to the nutrition interventions. This would align with a Brazilian study showing that early childhood diarrhea was associated with impaired fluency of language (54), and with an Haitian study where a link between nutritional factors (breastfeeding and complementary feeding frequencies, dietary diversity, egg and oil intake) and reduced infectious diseases improved language outcomes (55). Giving that early language development predicts future speech, grammar, reading, academic achievement and intelligence (56), our study provides evidence that our feasible intervention may have long-term benefits for children in these communities.

### Importance of Non-intervention Realities (MMN and Pets) on Child Development

An unexpected finding was that higher number of pets was associated with increased risk of motor and social/cognitive delay. Comparisons between communities showed that IC children had more dogs and cats at their homes, whereas CC children were more frequently exposed to vermin. Both pets (57) and vermin are vectors of infectious diseases (58–61). A study in Argentina identified that a greater number of people and animals (dogs, cats, chickens) in the home was associated with a greater infestation and abundance of disease-transmitting insects (61), but the association of the presence of pets with increased risk of areas of developmental delay has not been reported before. Domestic animal control may constitute a neglected preventable risk factor for child's health.

### Lessons Learned

This project recognized that mothers from the communities who were trained as CHPs were effective agents of social change. Wawa Illari taught us the value of empowering mothers independent of their educational level with tools that benefit themselves and their families.

Working with people in disadvantaged communities requires empathy, trust and flexibility. We learned that some community processes, such as CHPs' and caregivers' learning rhythms and creating trust and bonds with the community cannot be forced or rushed. The “step-by-step” learning system was key for achieving effectiveness in the practical application of concepts in gardens, hygiene, balanced meal preparation and appropriate caregiver child interaction.

Methodologies and field delivery systems need to be adapted to local capacities and field realities. Our monitoring and learning system revealed the importance of ongoing adaptation to changing situations including the inclusion of new institutional partners and implementing delivery mechanisms to reach intervention families.

There were situations of intra-family violence that discouraged CHPs from approaching violent households. The CHP, with our help, was able to understand that each family was a complex world. By listening to, learning from and adapting to CHPs and families' learning rhythm, schedules and routines we managed to implement a highly personalized and impactful project.

In order to reach the most vulnerable, we had to work within a parallel informal economy, weakly functioning institutions, and lack of basic water and sanitation infrastructure. Given that some communities lived in territories under no official state jurisdiction, they were not covered by legal benefits. Although out of the scope of our intervention, we realized that without local policies that improve essential community services, no intervention would be sufficiently effective for children to achieve their full potential.

### Strengths and Limitations

We were able to demonstrate an impact on food security, frequency of diarrheal and respiratory infections, and on language development. Indirect impacts of improving nutrition and caregiver-child interactions were observed through changes toward healthier feeding practices with increased intake of nutrients that are essential for child development, the treatment of parasitic infections and the support of MINSA policies regarding frequent growth and development follow up and intake of MMN. However, the 12-month timeframe of intervention may have been too short to demonstrate significant changes in traditional anthropometry indicators.

Initial differences between IC and CC made it more challenging to attribute changes directly to the intervention.

The Wawa Illari project was developed amid adverse circumstances, where frequent changes of health authorities at national and regional levels during the time of the project made administrative processes difficult. Political instability precluded us from reaching and making agreements with health authorities at the national level. Fortunately, constant communication with CHPs, support from local health authorities and health personnel, and family commitment to the methodology facilitated the resumption of activities and the continuity of the project.

## Conclusion

Through this multisector intervention that included home gardens, conscious nutrition workshops, and parenting skill development, we were able to demonstrate improvement in food security and language development in young children within a 12 months period, despite unfavorable field and institutional conditions. The success was in part attributed to on-site adaptations to adjust to changing local circumstances. The study highlights the value of framing indictors to the broad spheres of early motor, social/cognitive and language development. Importantly, we showed that training CHPs using the “step-by-step” learning system was effective for helping them to learn key messages and also to have a methodology for delivery of health-related knowledge and skills to caregivers in a way that allowed them to integrate activities into their daily life.

## Data Availability Statement

Given the highly sensitive local political situation and in order to protect the privacy of our vulnerable population, the datasets for this article are not publicly available. Participants did not sign informed consent that data will be publicly available, neither was this possibility discussed with Ethical Boards in Peru or Canada. Requests to access the datasets should be directed to Dr. Kristine G. Koski (kristine.koski@mcgill.ca).

## Ethics Statement

The studies involving human participants were reviewed and approved by Institutional Committee on Research Ethics of the National Institute for Child Health (registration OEAIDE-02997-2017), Lima, Peru. Written informed consent to participate in this study was provided by the participants' legal guardian/next of kin. Project received secondary approval by the Research Ethics Board at McGill University (REB File #144-0817).

## Author Contributions

DG-F, AM, VT, KK, and MS participated in study design. DG-F, AM, KK, MS, and CG participated in adaptation of questionnaires and acquisition of ethical approvals. DG-F coordinated data collection, ran statistical analyses, and performed statistical analyses. AM led field coordination and implementation including continuous training and monitoring of CHPs in the three methodologies, field accounting, and administration. AM and FH led the ICDP methodology. FH supported field coordination and implementation and helped with administrative field activities. IP participated in recruitment of CHPs, trainer of trainers for gardens' intervention, implemented the planting of fruit trees, and obtained additional funding. EG did training of trainers in conscious nutrition. NA provided continuous advice to local ICDP activities. VT did the international administration of the project and directly involved in principal and supplementary funding acquisition. SV facilitated introductory access to communities and the recruitment process. SH was the liaison-nurse with Pachacámac health authorities and verified technical accuracy of data collection. BR prepared logistics, analysed, and reported data on intestinal parasites. CG provided pediatric advice. DG-F, IP, AM, FH, VT, MS, and KK wrote the paper. All authors read and approved the content of the paper.

## Conflict of Interest

The authors declare that the research was conducted in the absence of any commercial or financial relationships that could be construed as a potential conflict of interest.

## Footnotes

^1^Contact information on “step-by-step” training: sofia.mazzini@gmail.com.

## Abbreviations

CC: control communities
CHPs: community health promoters
CPR: rural population centers
IC: intervention communities
HCAZ: head circumference for age Z-scores
ICDP: International Child Development program
LAZ: length for age Z-scores
MINSA: Peruvian Ministry of Health
MMN: multiple micronutrients
PAHO: Pan-American Health Organization
UIGV: Inca Garcilaso de la Vega University
WAZ: weight for age Z-scores
WLZ: weight for length Z-scores.
