# Supplementary material for: A Multi-Sectoral Approach Improves Early Child Development in a Disadvantaged Community in Peru: Role of Community Gardens, Nutrition Workshops and Enhanced Caregiver-Child Interaction: Project “Wawa Illari”
Source: Front Public Health. 2020 Nov 6;8:567900. doi: 10.3389/fpubh.2020.567900 (PMC7681241; doi:10.3389/fpubh.2020.567900)

Supplementary Material

1 Supplementary Tables

Table S1 List of foods commonly consumed in the region and for which intake across evaluations was assessed.

**Food**
Mark with a circle the pertinent item
Meat, chicken, fish, eggs, pork, turkey, tuna, seafood, guinea pigs
Oil vegetable, margarine, butter, butter, lard
Quinoa, wheat, amaranth, kanihua, barley, corn
Bread, noodles, biscuits
Milk, cheese, yogurt
Lentils, beans, broad beans, green peas, chickpeas
Spinach, swiss chard, broccoli, basil, cilantro, parsley, black mint
Tomato, carrots, pepper, pumpkin
Potato, sweet potato, cassava, 'maca', 'olluco', 'oca'
Papaya, apples, bananas, pineapple, peaches, grapes, granadilla, mango, orange, pear, lemon, cherimoya, passion fruit, sapodilla, plums
Peanuts, walnuts, almonds, chestnuts.
Ham, sausages
'Chicha', barley, emollients, tea, soft drinks, juice box, natural juices (made of fruits)
NAN Milk, S26, Enfamil, Similac
Cereal (Cerelac, Nestum)
Gerber, Nestlé Porridge

Table S2 Child development milestones by age group adapted to area of development.

Age	Motor	Social/Cognitive	Language
**<1 month**	Moro reflex<br>Suction reflex<br>Lying face up with arms and legs flexed and head sideways<br>Close hands		Blinking reflex
**1-2 months**	Alternate kicking<br>Opens hands	Social smiling	Uses voice to make sounds (vocalizing)
**2-4 months**	Rises his or her head	Looks at your face,<br>Tracks an object at mid-range	Reacts to sound
**4-6 months**	Holds objects	Responds to the examiner	Makes sounds
**6-9 months**	Brings objects to his/her mouth, actively changes position (rolls over)	Reaches for a toy	Locates the source of a sound
**9 -12 months**	Transfers objects from hand to hand, sits without support	Plays peek-a-boo	Duplicates syllables
**12 - 15 months**	Uses thumb and index finger to pick up small objects (pincer grasp)<br>Takes steps with support (cruising)	Imitates gestures	Babbles
**15-18 months**	Takes steps without support	Makes gestures on request<br>Places block in a cup	Says 1 word
**18-24 months**	Takes steps backwards	Identifies 2 objects<br>Scribbles spontaneously	Says 3 words
**24-28 months**	Gets undressed<br>Kicks a ball	Builds a 3-block tower	Points to 2 pictures
**28-34 months**	Gets dressed with supervision<br>Jumps on both feet	Builds a 6-cube tower	Makes 2-word sentences
**34-40 months**	Throws a ball	Reproduces a vertical line	Says a friend's name<br>Recognizes two actions
**40-46 months**	Puts on an undershirt<br>Moves his thumb while making a fist<br>Stands on each foot for 1 second		Understands two adjectives

2 Supplementary Figures

Figure S1 Likert scale used for caregiver-child interaction self-evaluation of caregivers, applied at the end of the intervention.

Community health promoters asked caregivers how they would currently qualify their attitudes reflecting each ICDP Guideline with options being: very low, low, medium, a lot, and very much. They asked the same question for how their attitudes were before ICDP workshops. Scores were marked as dots in the graph, with different colors for before and after. Dots were linked to create two lines that clearly showed caregivers and CHPs the guides in which there was an improvement and those that needed more work.

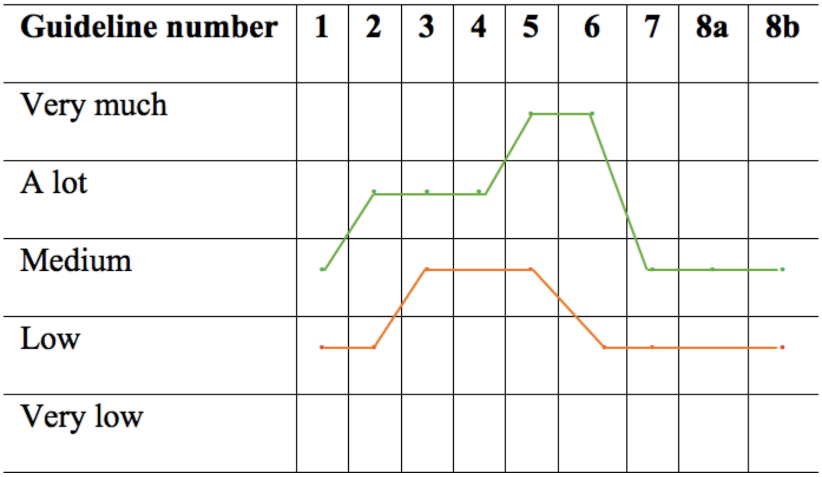

Guideline 1: the caregiver caresses, kisses, hugs, comforts the child
Guideline 2: demonstrates interest in what the child does, is sensitive to child's intentions, plays what the child proposes
Guideline 3: speaks to the child, smiles at him, looks at him in the eye, communicates at child's level of understanding, uses empathic tone of voice
Guideline 4: praises what the child does well, encourages him
Guideline 5: attracts his attention, joins child's interests
Guideline 6: names objects, colors, counts numbers
Guideline 7: gives explanations about the surroundings, makes comparisons, sings songs, tells stories
Guideline 8a: when correcting the child, the caregiver presents options, provides explanations and is firm
Guideline 8b: when the child tries to do something new, the caregivers accompanies the child in what he does, explains step by step, lets him try his best, provides support without interfering and accompanies child to achieve child's goal.

Figure S2 Age (months) distribution of children by gender in control and intervention communities.

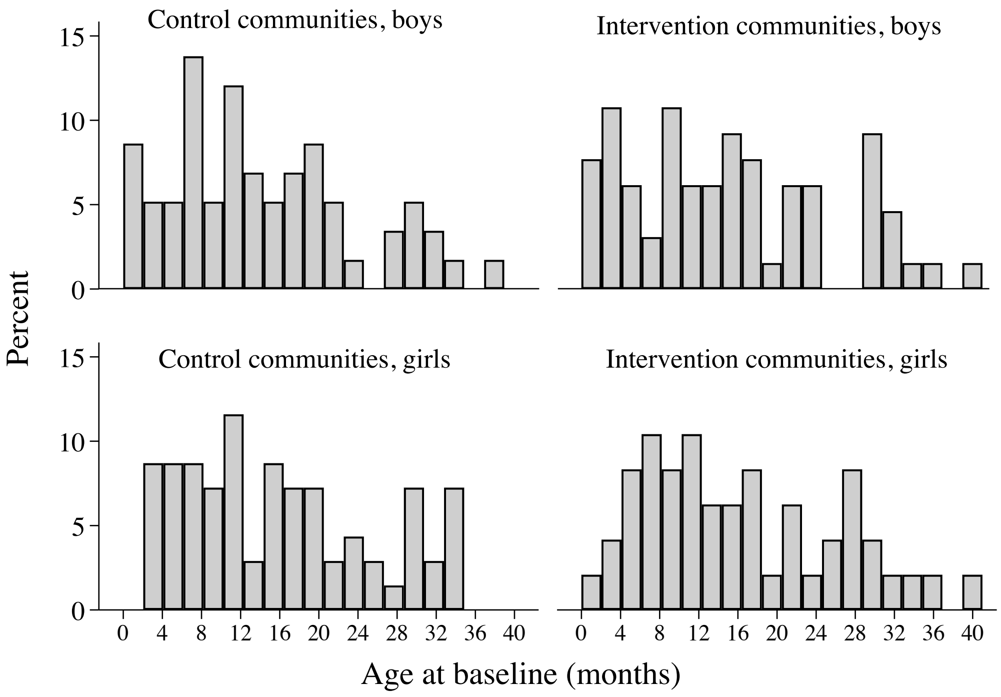

Figure S3 Box plots showing the number of foods taken by children as referred by caregivers at baseline and two post-intervention evaluations. The bottom and top of each box represent the 25th and 75th percentiles respectively; the horizontal line inside the box represents the median. Whiskers show the minimum and maximum values and dots represent outside values. The number of foods eaten by children was increased over time within both IC and CCs (P<0.0001), and children from the control community ate a higher variety of foods compared with children from the intervention community (P<0.0001).

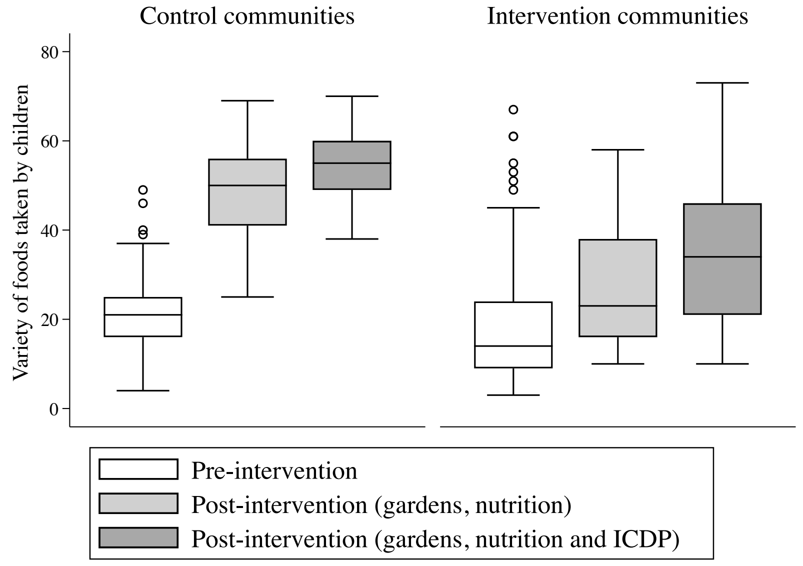

Figure S4 Mean number of (A) visits to the doctor due to illness, (B) episodes of diarrhea and (C) episodes of respiratory infections in the last month, at baseline, 8 months (after garden-nutrition interventions) and 12 months (after garden-nutrition-ICDP interventions) in control and intervention communities. 'A' or 'a' denote a significant higher mean than 'B' or 'b' (p= 0.0001).

A

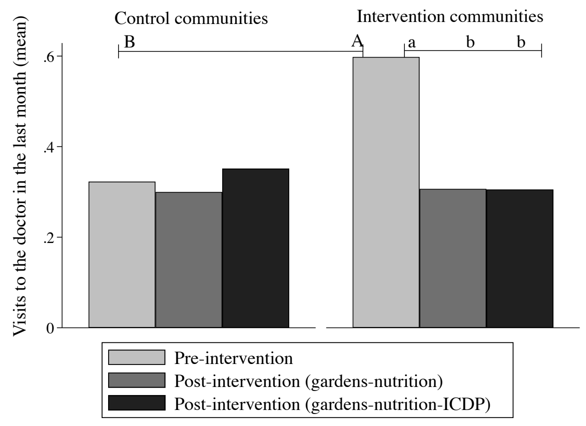

B

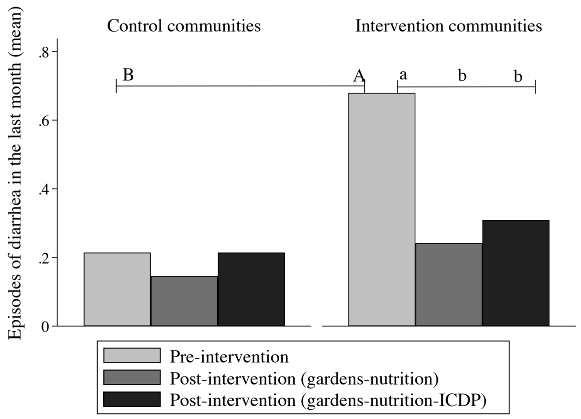

C

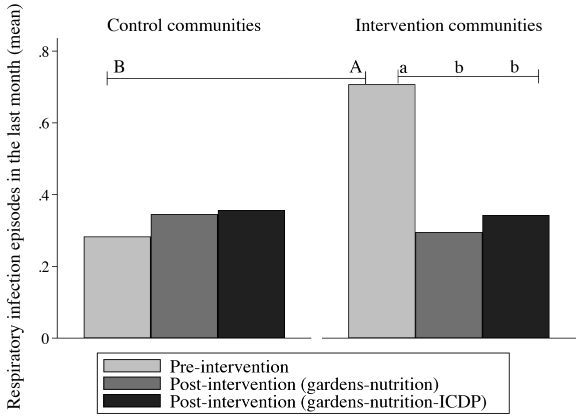

Supplement: Supplementary file 1 [file Data_Sheet_1.PDF]
